# Aero-Engine Remaining Useful Life Prediction Based on Bi-Discrepancy Network

**DOI:** 10.3390/s23239494

**Published:** 2023-11-29

**Authors:** Nachuan Liu, Xiaofeng Zhang, Jiansheng Guo, Songyi Chen

**Affiliations:** College of Equipment Management and UAV Engineering, Air Force Engineering University, Xi’an 710051, China; zhxfzhxf1@sina.com (X.Z.); amisc1@163.com (J.G.); sy931124@163.com (S.C.)

**Keywords:** remaining useful life prediction, domain adaptive regression, maximum classifier discrepancy, local maximum mean discrepancy

## Abstract

Most unsupervised domain adaptation (UDA) methods align feature distributions across different domains through adversarial learning. However, many of them require introducing an auxiliary domain alignment model, which incurs additional computational costs. In addition, they generally focus on the global distribution alignment and ignore the fine-grained domain discrepancy, so target samples with significant domain shifts cannot be detected or processed for specific tasks. To solve these problems, a bi-discrepancy network is proposed for the cross-domain prediction task. Firstly, target samples with significant domain shifts are detected by maximizing the discrepancy between the outputs of the dual regressor. Secondly, the adversarial training mechanism is adopted between the feature generator and the dual regressor for global domain adaptation. Finally, the local maximum mean discrepancy is used to locally align the fine-grained features of different degradation stages. In 12 cross@-domain prediction tasks generated on the C-MAPSS dataset, the root-mean-square error (RMSE) was reduced by 77.24%, 61.72%, 38.97%, and 3.35% on average, compared with the four mainstream UDA methods, which proved the effectiveness of the proposed method.

## 1. Introduction

Aero engine prognostics and health management (PHM) is an indispensable technology to enhance production dependability, operational safety, equipment maintenance effectiveness, and cost efficiency [1,2,3]. The remaining useful life (RUL) prediction is a crucial issue in the PHM sector and has great research value [4,5]. The system maintenance process can be considerably optimized by an accurate RUL prediction, which can assess the system’s health and assist users in developing reasonable maintenance plans [6,7].

The existing RUL prediction approaches are roughly divided into physical-model-based and data-driven approaches. The standard physical-model-based prediction method struggles to provide an accurate deterioration model because of aero engines’ complicated structure, the mathematical model’s severe nonlinearity, and the close coupling between sensor data [8]. In contrast, data-driven prediction methods based on a large amount of sensor data primarily rely on the corresponding intelligent algorithms to learn and characterize the degradation process of the system. These practical methods do not necessitate a deep understanding of the system’s internal workings or the intricate degradation mechanism [9]. Notably, numerous data-driven RUL-based prediction techniques surfaced in response to the quick development of sensor technologies.

Nevertheless, data-driven methods have some limits for the RUL prediction in industrial applications due to the following reasons: (1) the modeling process requires a large number of labeled datasets, and collecting enough labeled data is often complicated in many practical applications [10,11]; (2) it is assumed that offline training data and online test data come from the same feature space and obey the same distribution. However, in actual prediction tasks, there are invariably domain disparities between training and test data due to differences in the equipment operating circumstances and failure modes [12,13,14].

In order to improve the generalization ability of the model under different operating conditions and failure modes, domain adaptation (DA)-based transfer learning (TL) models were widely developed and applied to cross-domain RUL prediction tasks [15], which aim at extracting domain-invariant features between the source and target domains by using labeled samples from the source domain and a small number of unlabeled samples from the source domain to achieve an online RUL prediction in the target domain. Transfer learning is generally split into adversarial transfer learning and feature-based transfer learning. In order to minimize the distributional differences among features, the core of feature-based approaches entails mapping cross-domain data to a common feature space [16]. For example, Sun et al. proposed a sparse self-encoder-based deep migration learning network. They investigated three migration strategies, namely, weight migration, feature migration, and weight updating, to achieve an RUL prediction for machine tools [17]. Mao et al. used transfer component analysis (TCA) to sequentially adjust the features of the target bearing from those of the auxiliary bearings and then used the modified features to predict the RUL [18]. The adversarial-based approach draws on generative adversarial networks and consists of generative and discriminative models. The generative model extracts features from the source and target domains, making the discriminative model unable to distinguish which domain the extracted features come from. In contrast, the discriminative model maximally distinguishes which domain the extracted features come from, and the two play with each other to realize cross-domain feature migration. For example, Costa et al. combined a domain-adversarial neural network (DANN) with LSTM to reduce the distributional differences in cross-domain RUL prediction [19]. Ragab et al. proposed a comparative adversarial domain adaptive (CADA) method for the cross-domain RUL prediction of aero engines, which developed an adversarial domain adaptive architecture with contrast loss while learning domain-invariant features [20].

**Figure 1:**
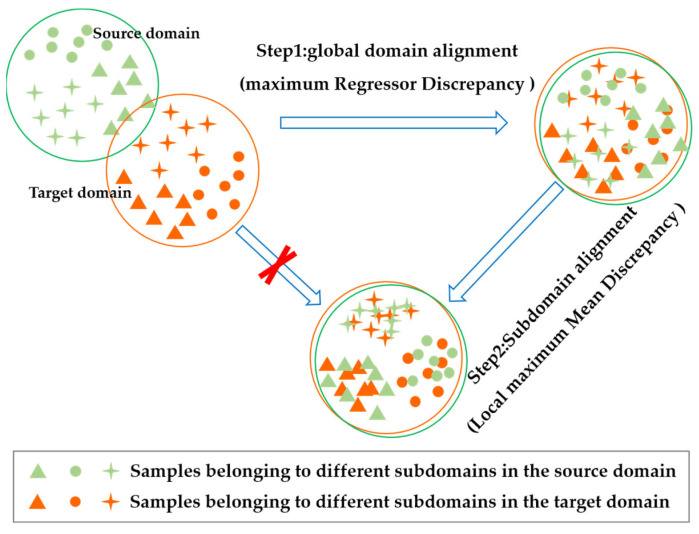
The schematic of global alignment and sub-domain alignment.

Despite some success in extracting domain-invariant features using the migration learning techniques mentioned above, it is essential to note that these issues still exist: (1) The sensor data of complex systems collected under different operating conditions and failure modes have significant distributional differences, and sensor data at multiple degradation stages also have different distributions. Therefore, solely adopting the global domain adaptation (GDA) technique could confuse the fine-grained features between the sub-domains denoted by various degradation stages [7], lowering RUL prediction performance. Furthermore, if the sub-domain adaptation (SDA) is directly carried out, the sub-domains might not be aligned because of the significant distributional variations between the source and target domains, which are shown in Figure 1. (2) Most of the current adversarial-based transfer learning techniques call for introducing an auxiliary model (discriminator) for the domain alignment, isolating the domain adaptation task from the prediction goal, and adding to the computational burden. (3) Under particular tasks, domain discriminators cannot discover or process target samples with significant domain shifts, as they can only classify features.

This paper proposes an aero-engine RUL prediction model based on a bi-discrepancy network (BDnet) to solve the above issues. First, target samples with significant domain shifts are identified by maximizing the regressor discrepancy without introducing additional models. Then, domain-invariant features are searched for based on the adversarial relationship between the feature generator and the dual regressor to achieve the GDA. Second, the fine-grained features of multiple degradation stages are extracted by the local maximum mean discrepancy (LMMD) to achieve the SDA. In particular, the SDA is performed on top of the GDA, which helps resolve the issue that direct sub-domain adaptation is ineffective due to the significant disparity in distribution between the source and target domains. Finally, 12 cross-domain prediction tasks from the aviation turbofan engines’ C-MAPSS dataset are used for evaluation. The outcomes demonstrate that the RUL prediction model based on the BDnet outperforms various popular feature-based transfer learning algorithms and adversarial-based transfer learning techniques.

The main contributions of this article are summarized as follows.

- First, the maximum regressor discrepancy (MRD) is proposed to detect and handle target samples with significant domain shifts without introducing additional models to achieve the GDA.
- Second, the local maximum mean discrepancy (LMMD) is designed to capture fine-grained information from multiple degradation stages, from which the domain-invariant features can be better learned to promote RUL prediction performance.
- Finally, the proposed BDnet-based RUL prediction approach is evaluated by an aircraft turbofan engine dataset under cross-domain conditions. The research results denote that the BDnet-based approach is better than some deep learning approaches without transfer learning and conventional transfer learning approaches.

The remainder of this paper is organized as follows. Section 2 discusses the theoretical background of MRD and the local maximum mean discrepancy in detail. Then, Section 3 discusses the RUL prediction method based on the BDnet in detail. Section 4 shows the results of the case study. Finally, Section 5 concludes this paper.

## 2. Theoretical Background

### 2.1. Maximum Regressor Discrepancy

In this paper, we draw on the concept of the maximum classifier discrepancy (MCD) [21] and propose the maximum regressor discrepancy to detect target domain samples that are significantly offset from the distribution of the source domain samples, as well as utilizing an adversarial training mechanism to gradually achieve the global domain alignment.

The distributional discrepancies between the source and target domain, in MCD’s opinion, may result in either excellent or unsatisfactory classification results if the network trained in the source domain is applied straight to the target domain. The target domain samples with subpar classification outcomes more accurately denote the domain variations. Two separate classifiers, $F_{1}$ and $F_{2}$, are introduced to detect and process these samples. If there is a significant variation in the classification outcomes of the two classifiers for the same sample, the sample is deemed to have a low confidence level and requires retraining.

The specific training process Is as follows:

First, the cross-entropy loss function is used to train the feature generator, G, and classifiers, F_1_ and F_2_, to correctly classify the source samples, which can be expressed as

(1) $$\begin{array}{c} \underset{G,F_{1},F_{2}}{\min}L\left(X_{S},Y_{S}\right)\\ L\left(X_{S},Y_{S}\right)=-E_{\left(x_{S},y_{S}\right)∼\left(X_{S},Y_{S}\right)}\sum_{k=1}^{K}I_{\left[k=y_{S}\right]}\log p\left(y|x_{S}\right) \end{array}$$

where $x_{s},x_{t}$ denote a labeled source data and a corresponding label, $y_{s}$, drawn from a set of labeled source data $\left\{X_{s},X_{t}\right\}$, respectively. K denotes the number of categories, $I_{\left[k=y_{s}\right]}$ denotes the one-hot vector of labels, and $p\left(y|x_{s}\right)$ denotes the K-dimensional probabilistic outputs for the input, $x_{s}$.

Second, on the basis of guaranteeing the classification ability of the model, the feature generator, G, is fixed, and the difference loss function is used to train two different classifiers, F_1_ and F_2_, to detect the samples with large domain shifts, which can be expressed as

(2) $$\begin{array}{c} \underset{F_{1},F_{2}}{\min}L\left(X_{S},Y_{S}\right)-L_{adv}\left(X_{t}\right)\\ L_{adv}\left(X_{t}\right)=E_{x_{t}∼X_{t}}\left[\frac{1}{K}\sum_{k=1}^{K}\left|p_{1}^{}\left(y|x_{t}\right)-p_{2}^{}\left(y|x_{t}\right)\right|\right] \end{array}$$

where $x_{t}$ denotes an unlabeled target image drawn from the unlabeled target images, $X_{t}$. $p_{1}\left(y|x_{t}\right),p_{2}\left(y|x_{t}\right)$ denote the K-dimensional probabilistic outputs for the input, $x_{t}$, obtained by $F_{1}$ and $F_{2}$, respectively.

Finally, the feature generator, G, is trained to minimize the classifier discrepancies, as shown in Equation (3).

(3) $$\underset{G}{\min}\;L_{adv}\left(X_{t}\right)$$

**Figure 2:**
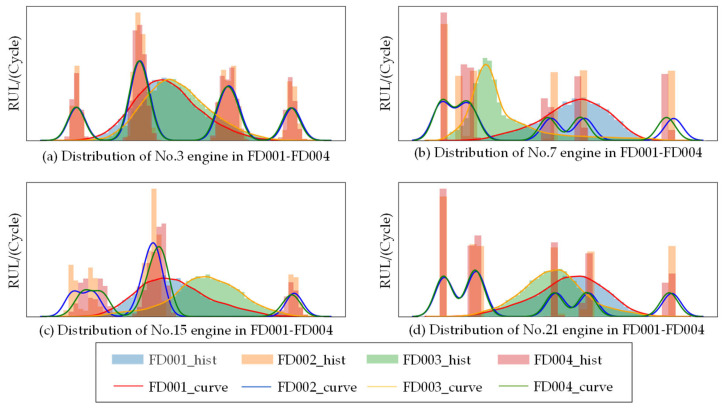
The difference in distribution for each C-MAPPS dataset.

Unlike MCD, MRD consists of a feature extractor, F, and two regressors, $\hat{R},\tilde{R}$, with identical structures. The feature extractor takes the labeled source domain data, $D_{S}={\left\{\left(X_{i}^{S},y_{i}^{S}\right)\right\}}_{i=1}^{N_{S}}$, and the unlabeled target domain data, $D_{T}={\left\{\left(X_{j}^{T}\right)\right\}}_{j=1}^{N_{T}}$, as inputs to generate high-level feature denotations. However, the significant cross-domain difference results in a feature distribution mismatch. Figure 2 shows the data distribution of four randomly selected sensors in four subsets of the C-MAPSS dataset. Among them, the data distributions of FD001 and FD003 are more similar, and the data distributions of FD002 and FD004 are more similar, which are mainly related to the failure modes and operating conditions of the dataset. However, the data distribution of the same sensor in the four subsets is significantly different. Therefore, the network parameters learned from single-source domain data cannot directly and accurately predict the target domain data. In order to align the source and target domain features, this paper uses the dual regressor as a discriminator to detect target domain samples with significant domain shifts. Suppose the dual regressor gives widely differing predictions for the same target domain sample. In this case, it is considered a domain-inconsistent sample with high confidence. At this point, training the feature generator can reduce this inconsistency. Unlike the classification task, since the labeled domain of the regression task is continuous and infinite, it is impossible to measure the discrepancies among the target domain samples by measuring the error between the conditional probability outputs of the two networks.

Considering the data imbalance problem, this paper utilizes the Tversky coefficient to quantify the difference between the dual regressors; however, the non-differentiable nature of the Tversky coefficient leads to the problem of updating the parameters of the network, so a differentiable form of the Tversky coefficient is proposed and is given by

(4) $$\begin{array}{ll} L_{tv} & =\sum_{i=1}^{N_{T}}\frac{\sum_{d}\left({\hat{h}}_{i}^{T}⊗{\tilde{h}}_{i}^{T}\right)}{\sum_{d}\left[{\hat{h}}_{i}^{T}⊗{\tilde{h}}_{i}^{T}+\alpha \left({\hat{h}}_{i}^{T}-{\hat{h}}_{i}^{T}⊗{\tilde{h}}_{i}^{T}\right)-\beta \left({\tilde{h}}_{i}^{T}-{\hat{h}}_{i}^{T}⊗{\tilde{h}}_{i}^{T}\right)\right]}\\ & =\sum_{i=1}^{N_{T}}\frac{\sum_{d}\left({\hat{h}}_{i}^{T}⊗{\tilde{h}}_{i}^{T}\right)}{\sum_{d}\left[\left(1-\alpha -\beta \right){\hat{h}}_{i}^{T}⊗{\tilde{h}}_{i}^{T}+\alpha {\hat{h}}_{i}^{T}-\beta {\tilde{h}}_{i}^{T}\right]} \end{array}$$

where α=β=0.5 denotes that the same weight is given to both regressors.  ${\hat{h}}_{i}^{T}=concatenate\left({\hat{h}}_{i}^{1,T},{\hat{h}}_{i}^{2,T},{\hat{y}}_{i}^{T}\right)$ and ${\tilde{h}}_{i}^{T}=concatenate\left({\tilde{h}}_{i}^{1,T},{\tilde{h}}_{i}^{2,T},{\tilde{y}}_{i}^{T}\right)$ denote the combination of the characteristics of the different stages for the two regressors. ⊗ denotes the Hadamard product.

**Figure 3:**
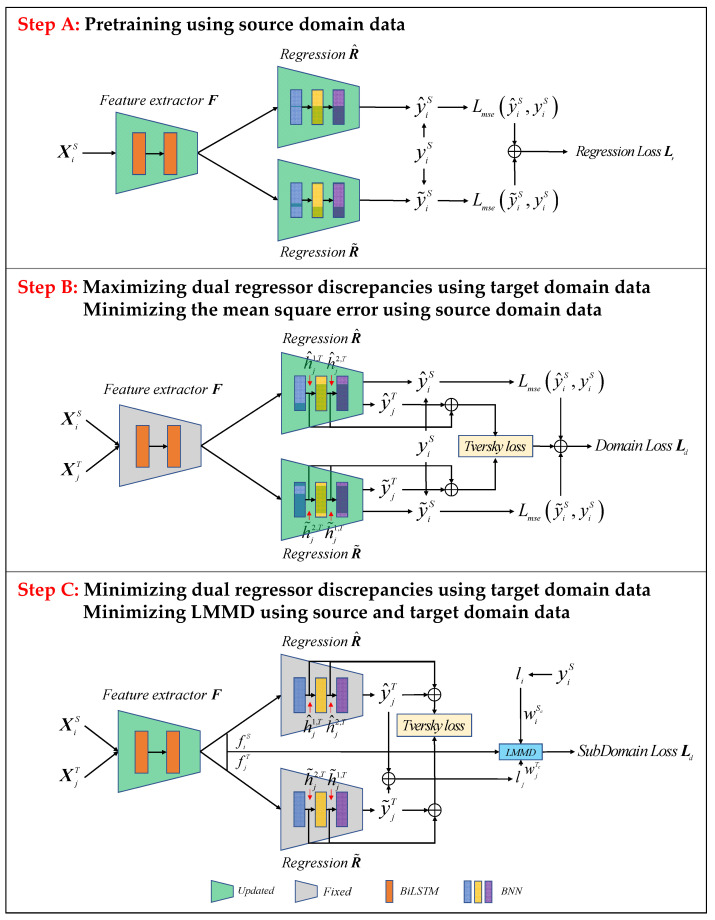
Training steps of bi-discrepancy network.

To facilitate the understanding of the process of implementing the GDA using a dual regression network, Figure 3 further demonstrates the three main steps.

- Step A: In order to ensure the prediction ability of the model for the source domain samples, the feature extractor and the dual regressors are pre-trained using the source domain data to minimize the mean squared error (MSE), as shown in Equation (5):

(5) $$\underset{F,\hat{R},\tilde{R}}{\min}L_{R}=\frac{\sum_{i=1}^{N_{S}}{\left({\hat{y}}_{i}^{S}-y_{i}^{S}\right)}^{2}}{N_{S}}+\frac{\sum_{i=1}^{N_{S}}{\left({\tilde{y}}_{i}^{S}-y_{i}^{S}\right)}^{2}}{N_{S}}$$

- Step B: In this step, we train the dual regressors as a discriminator for a fixed feature extractor. Since two regressors can easily produce inconsistent predictions for target instances with different sources, the prediction discrepancy means that two regressors involve different predictive abilities for the target samples. Thus, we utilize the prediction inconsistency to detect those target samples. Specifically, by training the dual regressors to increase the discrepancy, if the source and target regressors provide different predictions for the same target samples, we consider them as target domain samples with significant domain shifts. This step corresponds to Step B in Figure 3. We add a regression loss to the source samples to guarantee the predictive ability of the model for the source samples. We experimentally find that our algorithm’s performance significantly drops without this loss. The objective is as follows:

(6) $$\underset{\hat{R},\tilde{R}}{\min}L_{tv}+L_{R}$$

- Step C: We train the feature extractor to minimize the discrepancy for fixed dual regressors to move these target samples into the source support set. This step corresponds to Step C in Figure 3. This step denotes the trade-off between the extractor and the dual regressors. The objective is as follows:

(7) $$\underset{F}{\min}-L_{\mathrm{tv}}$$

### 2.2. Local Maximum Mean Discrepancy

Traditional DA methods usually use the maximum mean discrepancy (MMD) [22] to measure the discrepancy between the data distribution in the source domain and the data distribution in the target domain, and the MMD can be expressed as

(8) $$d_{H}\left(p,q\right)={\left\|E_{x∼p}\left[\phi \left(x^{S}\right)\right]-E_{x∼q}\left[\phi \left(x^{T}\right)\right]\right\|}_{H}^{2}$$

where H denotes the reproducing kernel Hilbert space (RKHS) defined by the salient kernel. k means the kernel function relation $k\left(x^{S},x^{T}\right)=\langle \phi \left(x^{S}\right),\phi \left(x^{T}\right)\rangle$. ϕ defines the transformation from the initial data to the RKHS. 〈·〉 means the inner product operation. In this paper, based on MMD, considering the intrinsic relationship of each sub-domain in the different degradation stages of the aero engine, the local maximum mean discrepancy (LMMD) is utilized to denote the discrepancy in the data distribution between the source domain and the target domain, as shown in Equation (9):

(9) $$d_{H}\left(p,q\right)=E_{c}{\left\|E_{x∼p^{c}}\left[\phi \left(x^{S}\right)\right]-E_{x∼q^{c}}\left[\phi \left(x^{T}\right)\right]\right\|}_{H}^{2}=\frac{1}{N_{c}}{\sum_{c=1}^{N_{c}}\left\|\sum_{x_{i}^{S}\in D^{S}}^{}w_{i}^{S_{c}}\phi \left(x_{i}^{S}\right)-\sum_{x_{j}^{T}\in D^{T}}^{}w_{j}^{T_{c}}\phi \left(x_{j}^{T}\right)\right\|}_{H}^{2}$$

where $p^{c}$ and $q^{c}$ denote the distribution of subdomains belonging to category c in the source and target domains, respectively. $w_{i}^{S_{c}}$ and $w_{j}^{T_{c}}$ denote the weights of the i-th source domain sample and the j-th target domain sample belonging to category c, respectively, besides $\sum_{i=1}^{N_{S}}w_{i}^{S_{c}}=1$ and $\sum_{j=1}^{N_{T}}w_{j}^{T_{c}}=1$. Unlike the classification problem with finite-dimensional data labels, regression problems have infinite labels. Therefore, it is not possible to directly use LMMD. In this paper, we utilize the discretization strategy of the degradation process proposed by Zhang et al. [7] to discretize the whole degradation process into multiple degradation stages, and the i-th degradation stage can be expressed as

(10) $$l_{i}=round\left(\frac{RUL_{\max}-RUL_{t}^{i}}{RUL_{\max}}N_{c}\right)$$

where $RUL_{\max}=125$ is the threshold of the piecewise linear degradation model. In detail, the early operating stages of an aircraft turbofan engine can be considered healthy. When the RUL is less than a threshold, the turbofan engine begins to degrade [23]. $RUL_{t}^{i}$ is the actual remaining life of the i-th sample, and $N_{c}=10$ is the number of divided degradation stages. Therefore, for sample $x_{i}$, $w_{i}^{c}$ can be expressed as

(11) $$w_{i}^{c}=\frac{l_{i}^{c}}{\sum_{\left(x_{i},l_{i}\right)\in D}l_{i}^{c}}$$

where $l_{i}^{c}$ is the c-th element of the vector generated by $l_{i}$ after one-hot encoding. For the source domain samples, the actual RUL labels can be directly utilized to compute $l_{i}$ and, hence, $w_{i}^{S_{c}}$. However, for the target domain samples, there are no actual RUL labels used to compute $w_{i}^{c}$. Therefore, in this paper, we utilize a dual regression network for prediction and compute $y_{j}^{T}=\left({\hat{y}}_{j}^{T}+{\tilde{y}}_{j}^{T}\right)/2$ as the RUL labels of the target domain samples to obtain $l_{i}$, and, thus, $w_{j}^{T_{c}}$. Thus, Equation (9) can be rewritten as

(12) $$d_{H}\left(p,q\right)=\frac{1}{N_{c}}\sum_{c=1}^{N_{c}}\left[\sum_{i=1}^{N_{S}}\sum_{j=1}^{N_{S}}w_{i}^{S_{c}}w_{j}^{S_{c}}k\left(f_{i}^{S},f_{j}^{S}\right)+\sum_{i=1}^{N_{T}}\sum_{j=1}^{N_{T}}w_{i}^{T_{c}}w_{j}^{T_{c}}k\left(f_{i}^{T},f_{j}^{T}\right)-2\sum_{i=1}^{N_{S}}\sum_{j=1}^{N_{T}}w_{i}^{S_{c}}w_{j}^{T_{c}}k\left(f_{i}^{S},f_{j}^{T}\right)\right]$$

where $f^{S/T}=F\left(X^{S/T}\right)$ is the feature extracted by the feature extractor.

## 3. RUL Prediction Method Based on BDnet

Section 2 explains the details of implementing the BDnet to seek a domain-invariant representation for cross-domain regression. In this section, the prediction process of the RUL prediction method based on the BDnet is further explained. The RUL prediction can generally be divided into the offline training phase and the online prediction phase. For the offline training phase, firstly, the source domain data, $D_{S}={\left\{\left(X_{i}^{S},y_{i}^{S}\right)\right\}}_{i=1}^{N_{S}}$, and the target domain data, $D_{T}={\left\{\left(X_{j}^{T}\right)\right\}}_{j=1}^{N_{T}}$, are divided by using the time window to obtain the training data. Secondly, the mapping relationship between the source domain data and the RUL is constructed by the source domain data, $D_{S}$, and the GDA of the source and target domains is realized according to Equations (1)–(4). Finally, the network parameters are tuned using the LMMD to realize the SDA. For the online prediction stage, the partitioned target domain data are fed into the BDnet to obtain the mean and variance of the RUL prediction. Since the sensor values at the current moment in the aero engine degradation process are closely related to the degradation states at the time steps before and after, Bi-LSTM [24] is used as a feature extractor to extract the time dependence from the sensor data in this paper. Then, to improve the accuracy of the RUL and quantify the uncertainty of the prediction results, Bayesian neural networks (BNNs) [25] with Monte Carlo dropout inference are utilized as a regressor for uncertainty prediction. The pseudo-code of the RUL prediction method based on the BDnet is shown in Algorithm 1.**Algorithm 1** RUL prediction based on bi-discrepancy network**Input:** Train data $D_{S}={\left\{\left(X_{i}^{S},y_{i}^{S}\right)\right\}}_{i=1}^{N_{S}}$ in the source domain, 
Train data $D_{T}={\left\{\left(X_{j}^{T}\right)\right\}}_{j=1}^{N_{T}}$$\;\mathrm{in}\;\mathrm{the}\;\mathrm{target}\;\mathrm{domain},\;\mathrm{Test}\;\mathrm{data}\;D_{T}^{\mathrm{test}}={\left\{\left(X_{j}^{T}\right)\right\}}_{j=1}^{N_{T}^{\mathrm{test}}}$$\;\mathrm{in}\;\mathrm{the}\;\mathrm{target}\;\mathrm{domain},\;\mathrm{Maximum}\;\mathrm{number}\;\mathrm{of}\;\mathrm{training}\;epoch_{\max}$. 
1:Selection variable and swipe the time window to generate samples.
2:Initialize randomly the model parameters $\theta_{f},\theta_{\hat{R}},\theta_{\tilde{R}}$. 
3:Initialize epoch=1.
4:**while** $epoch\leq epoch_{\max}$ **do**

5:  Input $D_{S}$ to Bi-Discrepancy Network for calculating regression loss $L_{R}$ through Equation (2).
6:  Update $\theta_{f},\theta_{\hat{R}},\theta_{\tilde{R}}$ using the back-propagation method to minimize $L_{R}$.

7:  Input $D_{S}$ to Bi-Discrepancy Network for calculating regression loss $L_{R}$ through Equation (2) and input $D_{T}$ to Bi-Discrepancy Network for calculating dual regression discrepancy $L_{tv}$ through Equation (1).

8:  Update $\theta_{\hat{R}},\theta_{\tilde{R}}$ using the back-propagation method to minimize $L_{R}+L_{tv}$.

9:  Calculate source domain data’s degenerative level $l_{i}^{S}$ through Equation (7).

10:  Input $D_{T}$ to Bi-Discrepancy Network for calculating ${\hat{y}}_{j}^{T},{\tilde{y}}_{j}^{T}$.

11:  Calculate target domain data’s degenerative level $l_{j}^{T}$ with $RUL_{t}^{j}=\left({\hat{y}}_{j}^{T}+{\tilde{y}}_{j}^{T}\right)/2$ through Equation (7).
12:  Calculate source weights and target weights $w_{i}^{S_{c}},w_{j}^{T_{c}}$ of LMMD.
13:  Calculate LMMD loss $L_{lmmd}$ through Equation (9).
14:  Update $\theta_{f}$ using the back-propagation method to minimize $\left(-L_{tv}\right)+\lambda L_{lmmd}$.
15:  epoch→epoch+1.
16:**end while**
17:$\mathrm{input}\;D_{T}^{test}$$\;\mathrm{to}\;\mathrm{Bi}-\mathrm{Discrepancy}\;\mathrm{Network}\;\mathrm{for}\;\mathrm{calculating}\;{\hat{y}}_{j}^{T},{\tilde{y}}_{j}^{T}$ with the optimal model parameters.

where λ is a hyperparameter used to control the ratio between GDA and SDA.

## 4. Case Study

### 4.1. Dataset and Data Preprocessing

First, this paper uses the C-MAPSS dataset provided by the NASA-Ames Research Center [26], divided into four subsets, FD001~FD004, according to different operating conditions and failure modes. Each subset consists of a training set and a test set. The training dataset contains the whole lifetime data, and the testing dataset includes the initial degradation data within a period of time, which are used for model validation. The detailed setup is shown in Table 1. Since the four sub-datasets were obtained under different failure modes and operating conditions, the data distributions significantly differed. In this sense, the research objective of this paper is to train the BDnet only using the labeled source domain data under a certain operational condition and the unlabeled target domain data under another operational condition. Furthermore, the RUL prediction is implemented for the online target domain data.

The monitoring data for each flight cycle consist of 26 dimensions of feature data, where the first two dimensions denote the engine (unit) number and the cycle number. The following three dimensions are the flight conditions (flight altitude, Mach number, and throttle-stick solver angle), and the remaining 21 dimensions are the monitoring data. In addition, to prevent the redundancy and interaction of the multidimensional sensor data from adversely affecting the RUL prediction, this paper analyzes the degree of correlation among the sensors and the trend of degradation of each sensor over time to select the optimal sensor signal for the RUL prediction. The correlation and monotonicity measures can be expressed as follows [27].

(13) $$\begin{array}{l} c=\frac{\left|n\sum_{n}F_{t}\cdot t-\sum_{n}F_{t}\sum_{n}t\right|}{\sqrt{\left[n\sum_{n}F_{t}^{2}-{\left(\sum_{n}F_{t}\right)}^{2}\right]\left[n\sum_{n}t^{2}-{\left(\sum_{n}t\right)}^{2}\right]}}\\ m=\frac{\left|\sum_{n}\delta \left(F_{t+1}-F_{t}\right)-\sum_{n}\delta \left(F_{t}-F_{t+1}\right)\right|}{n-1} \end{array}$$

where *c* and m represent correlation and monotonicity, respectively. $F_{t}$ represents the feature value at cycle t. δ(⋅) represents the unit step function. n indicates the quantity of the signals. The range of both c and m is [0, 1]. *c* = 1 represents the complete correlation, and *m* = 1 represents the monotonic increasing or monotonic decreasing. Thus, sensors with a larger (c+m)/2 can better represent the changing trend and are selected. Take FD001 as an example: #2, #3, #4, #7, #8, #11, #12, #13, #15, #17, #20, and #21 are selected when the threshold is set to 0.75.

Moreover, the same sensor may have different measurements under different operating conditions. In order to reduce the effect of the operating conditions, the sensor data under each operating condition are min–max normalized so that the data size is limited to the range of [0, 1]. Finally, a time window with a window size of 30 and a step size of 1 is utilized to divide the raw data to generate the training and testing datasets.

### 4.2. Simulation Conditions and Parameter Settings

The hardware and software environment of the simulation is an NVIDIA GeForce RTX 3060 Laptop GPU, an Intel Core i7-11800 H CPU, 32 G RAM, Windows 11, and Python 3.7, based on the PyTorch framework. Manufacturer is Lenovo (Beijing, China).

The BDnet consists of three main parts: a feature extractor, a dual regressor, and a subdomain adaptor. The main parameters are defined as follows: the time window length is set to 30, the number of loops is 200, the early stop mechanism is set, the degeneracy inflection point is 125, the hidden layer dimension of the dual regressor is 32, and the optimizer adopts the Adam optimizer. In addition, the performance of the prediction model may be affected by the different batch sizes in the neural network, the learning rate of the feature extractor, the learning rate of the dual regressor, the dimension of the hidden layer of the Bi-LSTM, the number of layers of the Bi-LSTM, and the scaling factor. Therefore, it is essential to investigate the adaptability and robustness of the model for different tasks by adjusting the hyperparameters. The hyperparameter configuration is shown in Table 2.

**Figure 4:**
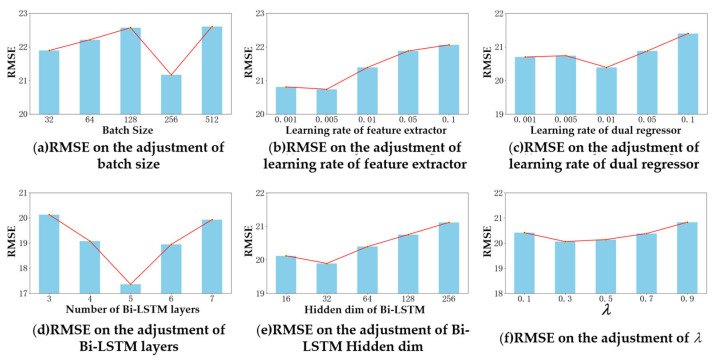
Experimental results with different hyperparameters for FD001–FD002 cross-domain prediction tasks.

Take FD001–FD002 as an example to discuss the influence of the hyperparameters on prediction performance, and the results are shown in Figure 4. As shown in Figure 4, the cross-domain prediction performance is best when the batch size is 256, the feature extractor learning rate is 0.005, the dual regressor learning rate is 0.01, the number of Bi-LSTM layers is five, the dimension of the Bi-LSTM hidden layer is 32, and λ is 0.3. The hyperparameter settings for all the cross-domain combinatorial research tasks are shown in Table 3.

### 4.3. Evaluation Indicators

In order to measure the predictive performance of the proposed model, the scoring function (SF) and root-mean-square error (RMSE) are used for quantitative assessment.

The SF is an asymmetric function often used to evaluate the performance of the remaining life prediction. For the remaining life prediction of an aero engine, the ahead prediction is better than the lagged prediction, so the lagged prediction is more severely penalized than the ahead prediction for the same error. The SF is defined as

(14) $$SF=\{\begin{array}{cc} \sum_{i=1}^{N}\left(e^{-\frac{{\hat{y}}_{i}-y_{i}}{13}}-1\right) & {\hat{y}}_{i}-y_{i}<0\\ \sum_{i=1}^{N}\left(e^{\frac{{\hat{y}}_{i}-y_{i}}{10}}-1\right) & {\hat{y}}_{i}-y_{i}\geq 0 \end{array}$$

Compared to the SF, the RMSE equally penalizes the over-advanced and lagged prediction errors. The RMSE is defined as

(15) $$RMSE=\sqrt{\frac{1}{N}{\sum_{i=1}^{N}\left({\hat{y}}_{i}-y_{i}\right)}^{2}}$$

where ${\hat{y}}_{i}$ and $y_{i}$ are the predicted and actual values of the i-th sample of the testing set, respectively. N is the number of samples.

### 4.4. Experimental Results Analysis and Discussions

**Figure 5:**
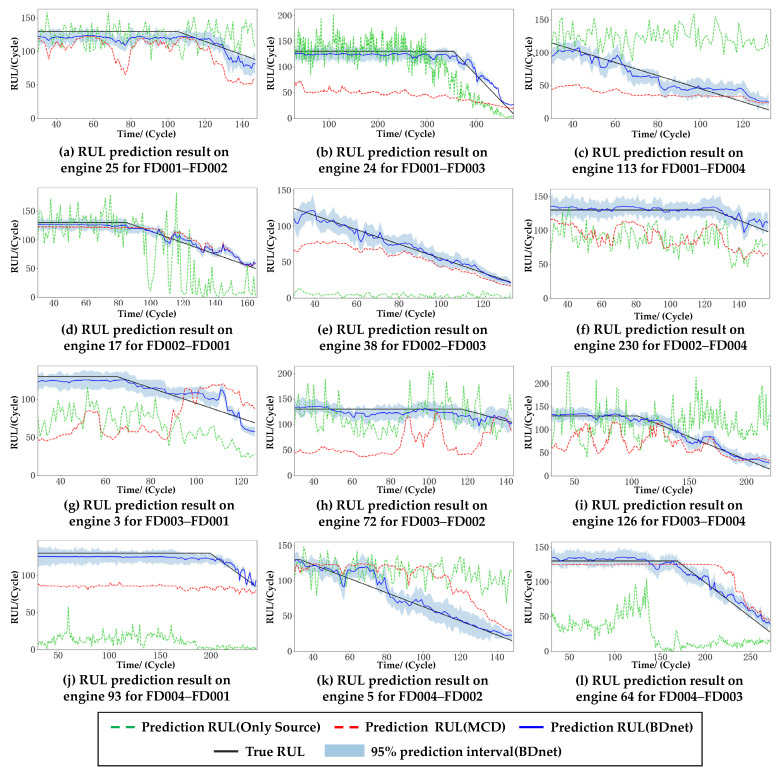
RUL predictions of three models for 12 randomly selecting engines coming from the target domain.

In order to validate the performance of the BDnet on the cross-domain prediction tasks, ablation experiments are first performed. The model that only utilizes the feature extractor and dual regressor directly trained with the source domain data on the target domain data is defined as model 1, which can be considered as the benchmark model for the RUL prediction problem. The model that uses the MCD is defined as model 2, and the model that uses both the MCD and the LMMD is defined as model 3. The three models’ predictions, as mentioned above, are compared with the real RUL. The models are trained using the source domain data and evaluated on the target domain. Since the C-MAPSS dataset has four subsets, 12 cross-domain prediction tasks are generated, and the results are shown in Figure 5.

First, model 3 gives the highest accuracy on all 12 cross-domain prediction tasks. Second, model 2 and model 3 are more stable and less fluctuating than model 1.

Taking FD001 as the source domain data, the cross-domain prediction results for the target domains of FD002, FD003, and FD004 are shown in Figure 5a–c, respectively. Since FD001 has a significant distributional discrepancy compared to FD002 and FD004, an apparent two-stage optimization structure can be seen, i.e., the distribution of the source and target domains is firstly brought closer by the GDA to learn the degradation trend of the target domain (red dashed line). Then, the prediction results are fine-tuned by the SDA to better fit with the real RUL (blue solid line). Although the data distributions of FD001 and FD003 are more similar, model 2 in Figure 5b gives the most significant prediction error compared to model 2 in Figure 5a,c. This is mainly because the maximum regressor difference is optimized by finding the difference features between the source and target domains. If the difference is slight, then multiple pieces of training may lead to overfitting. Therefore, we increase the degree of subdomain adaptivity by increasing the value of λ to achieve higher prediction accuracy.

Figure 5d–f demonstrate the cross-domain prediction results with the source domain as FD002 and the target domains as FD001, FD003, and FD004. Similar to the results with the source domain as FD001, the prediction accuracy is higher when the target domains are FD001 and FD003, and FD001 does not need subdomain adaptation because FD001 has only one failure mode compared to FD003.

Figure 5g–i demonstrate the cross-domain prediction results with FD003 as the source domain, and the prediction results of model 2 for all three tasks are significantly degraded, which is mainly due to the multi-fault modes. In addition, the degradation trend of the target domains, FD002 and FD004, is consistent with the real RUL. In contrast, the degradation trend of the target domain, FD001, shows a significant deviation from the real RUL. Thus, the λ of this group of experiments is increased in comparison with that of the previous two groups of experiments.

Like the last set of experiments, the source domain, FD004, also has two fault modes. However, the prediction results are better because FD004 has more training data and adequate feature extraction.

As shown in Table 4, model 1 has the worst prediction performance, and its prediction results heavily rely on the similarity between the source and target domain data distributions. For model 2, except for the four results of the RMSE and the two results of the SF (underlined) that are higher than their counterparts in model 1, the other results are better than their counterparts in model 1, and the result of the RMSE for FD002–FD001 in model 2 is the optimal result for the three models (bold). For model 3, all results are optimal for the three models, except for a result of the RMSE that is slightly higher than the corresponding result in model 2. Compared to model 1, the prediction model without domain adaptation, model 3 has an average reduction of 50.62% and 76.99% for the results of the RMSE and SF. Compared to model 2, with only global domain adaptation, model 3 has an average reduction of 35.16% and 47.66% for the results of the RMSE and SF. Therefore, in most cases, model 3 predicts better than model 1 and model 2, proving the effectiveness of the BDnet-based prediction model.

**Figure 6:**
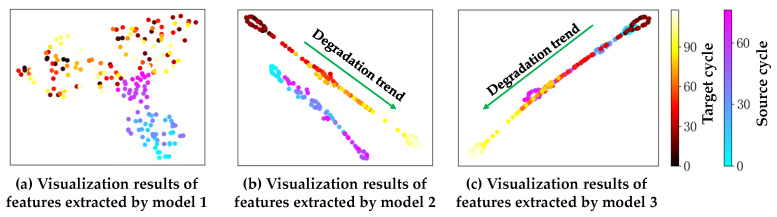
Visualization results of the learned features by three methods of No. 4 engine from source domain and No. 5 engine from target domain for FD001–FD002 cross-domain prediction tasks.

Figure 6 depicts the distribution of the feature vectors extracted by the three models to further validate the migration learning effect based on the BDnet prediction model (t-SNE is used in this work). For model 1, the representations of the features extracted from the source and target domains are more dispersed in two-dimensional space, and the degradation trend is not apparent enough, which further illustrates the discrepancy in the data distribution under different operating conditions and fault modes. With global domain adaptation, the degradation trend shown in Figure 6b is pronounced, and there is only a slight deviation in the distribution of features extracted from the two domains. Fine-tuned by subdomain adaptation, the health status of the two domains in model 3 are clustered in the same region, and the degradation processes are well-aligned. Thus, the visualization results intuitively validate the effectiveness of the BDnet-based prediction model. 

To assess the quality of the model in transferring the degradation patterns from a source to a target domain, the BDnet-based predictive model is compared with four mainstream UDA methods targeting the C-MAPSS dataset. TCA-DNN and CORAL-DNN are UDA methods that combine traditional machine learning algorithms with deep neural architectures. TCA [28] uses MMD to learn cross-domain migration components in the regenerative kernel RKHS to construct a feature space that minimizes the domain differences. CORAL [29] aligns the second-order statistical features of the distributions of the source and the target domains using linear transformations. LSTM-DNN [19] and CADA [20] are typical adversarial UDA methods. LSTM-DNN consists of a feature extractor, a RUL predictor, and a domain discriminator, whereas CADA adds a contrast loss estimation module to minimize adversarial loss.

**Figure 7:**
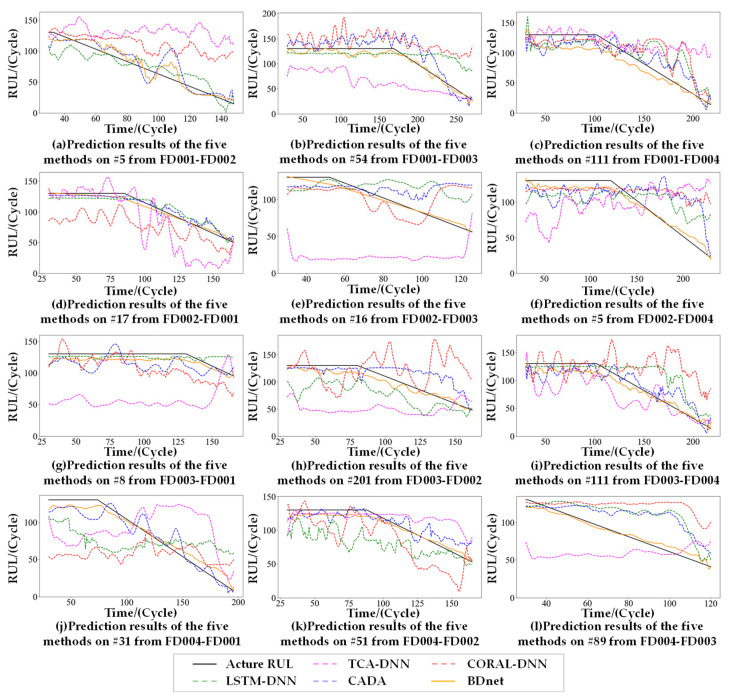
RUL predictions of five methods for 12 randomly selected engines coming from the target domain.

Twelve engines were randomly selected for comparison experiments on 12 cross-domain prediction tasks, and Figure 7 demonstrates the combined comparison results of the 12 engines for the five methods. The proposed method achieves good prediction performance compared to the four mainstream methods. TCA-DNN and CORAL-DNN are basically unable to learn the trend information of the degradation process. Although LSTM-DNN can learn the trend information of the degradation process, the prediction results significantly differ from the actual value due to the lack of local subdomain adaptation. In contrast, CADA obtained better prediction results by the contrastive loss module for fine-tuning, but, compared with the BDnet, CADA’s prediction results have greater volatility.

The comparative experimental results of the RMSE are shown in Table 5. The proposed method is better than TCA-DNN, CORAL-DNN, and LSTM-DNN on all 12 cross-domain prediction tasks, with only four tasks being slightly lower than CADA, with an average reduction of 77.24%, 61.72%, 38.97%, and 3.35% in the RMSE values compared to the four comparison methods.

The comparative experimental results of the SF are shown in Table 6. The proposed method is better than TCA-DNN [19], CORAL-DNN [19], and LSTM-DNN [19] on all 12 cross-domain prediction tasks, with six tasks being slightly lower than CADA [20], with an average reduction of 42.12% in the RMSE values compared to CADA.

In addition, the BDnet significantly improves the prediction performance of the larger domain offset prediction task while maintaining the performance of the smaller domain offset prediction task, proving the superiority of the BDnet without introducing other auxiliary models.

## 5. Conclusions

In this paper, an unsupervised domain adaptive regression method based on the BDnet was proposed to address the realistic problem of unlabeled samples in the aero engine RUL prediction task. Unlike previous adversarial DA methods that introduce discriminators for domain feature alignment, the BDnet achieves the GDA by maximizing the regressor difference to gradually detect and adjust the target domain samples with significant domain shifts, without introducing an additional model. In addition, to extract the fine-grained features of the aero engine at different degradation stages, the LMMD is utilized to achieve the SDA based on the GDA. Finally, experiments are conducted on 12 cross-domain prediction tasks to verify the effectiveness of the BDnet-based RUL prediction model in solving the unsupervised domain adaptive regression problem.

## Figures and Tables

**Table 1:** The description of aircraft turbofan engine dataset.

Dataset	Training Engines	Testing Engines	Fault Modes	Operational Conditions
FD001	100	100	1	1
FD002	260	259	1	6
FD003	100	100	2	1
FD004	249	248	2	6

**Table 2:** Hyperparameter configuration of BDnet.

Hyperparameter	Configuration
Batch size (bs)	32, 64, 128, 256, 512
Learning rate of the feature extractor (lr-F)	0.001, 0.005, 0.01, 0.05, 0.1
Learning rate of the dual regressor (lr-R)	0.001, 0.005, 0.01, 0.05, 0.1
Dimension of the hidden layer of the Bi-LSTM (hd)	16, 32, 64, 128, 256
Number of layers of the Bi-LSTM (nl)	3, 4, 5, 6, 7
Scaling factor (λ)	0.1, 0.3, 0.5, 0.7, 0.9

**Table 3:** Hyperparameters configuration under various cross-domain prediction tasks.

Task	Batch Size	Learning Rate of Feature Extractor	Learning Rate of Dual Regressor	Hidden Dim of Bi-LSTM	Number of Bi-LSTM Layers	λ
FD001–FD002	256	0.005	0.01	5	32	0.3
FD001–FD003	256	0.001	0.01	1	32	0.7
FD001–FD004	256	0.01	0.01	5	64	0.3
FD002–FD001	256	0.01	0.01	5	64	0.1
FD002–FD003	256	0.01	0.01	5	64	0.1
FD002–FD004	256	0.005	0.01	3	32	0.5
FD003–FD001	256	0.01	0.01	1	32	0.9
FD003–FD002	256	0.01	0.01	5	128	0.5
FD003–FD004	256	0.01	0.01	5	128	0.3
FD004–FD001	256	0.01	0.01	5	64	0.5
FD004–FD002	256	0.005	0.01	3	32	0.7
FD004–FD003	256	0.01	0.01	5	64	0.3

**Table 4:** Comparison of RMSE and SF for three models under various cross-domain prediction tasks.

Task	Model 1		Model 2		Model 3	
	RMSE	SF	RMSE	SF	RMSE	SF
FD001–FD002	41.11	35,853.06	28.17	4438.99	20.83	3563.29
FD001–FD003	31.98	5572.99	73.32	10,117.37	21.28	2189.71
FD001–FD004	38.38	10,130.34	35.14	7713.32	28.57	4003.32
FD002–FD001	70.73	6636.28	16.11	582.78	16.14	474.32
FD002–FD003	79.51	12,905.80	39.90	4793.32	22.65	2525.53
FD002–FD004	43.39	81,918.88	39.30	7003.99	31.88	8131.71
FD003–FD001	32.61	3875.72	54.98	3698.78	18.86	2725.45
FD003–FD002	45.71	48,222.12	49.48	16,427.39	21.67	5923.09
FD003–FD004	42.21	9802.89	36.73	8448.22	19.24	4102.11
FD004–FD001	98.75	72,967.48	38.53	7330.92	19.48	3535.12
FD004–FD002	32.62	8970.82	34.84	10,423.48	25.1	4070.49
FD004–FD003	101.79	18,233.97	20.61	609.38	13.74	549.18

**Table 5:** Comparison of RMSE for the proposed method against state-of-the-art approaches.

Task	TCA-DNN [19]	CORAL-DNN [19]	LSTM-DANN [19]	CADA [20]	BDnet
FD001–FD002	90.0	77.5	48.6	19.52	20.83
FD001–FD003	116.1	69.6	45.9	39.58	21.28
FD001–FD004	113.8	84.6	43.8	31.23	28.57
FD002–FD001	85.6	80.9	28.1	13.88	16.14
FD002–FD003	111.5	79.8	37.5	33.53	22.65
FD002–FD004	94.4	43.6	31.9	33.71	31.88
FD003–FD001	90.5	26.5	31.7	19.54	18.86
FD003–FD002	80.8	75.6	44.6	19.33	21.67
FD003–FD004	102.6	77.2	47.9	20.61	19.24
FD004–FD001	85.6	94.0	31.6	20.10	19.48
FD004–FD002	80.8	30.9	24.9	18.50	25.10
FD004–FD003	102.9	68.6	27.8	14.49	13.74

**Table 6:** Comparison of SF for the proposed method against state-of-the-art approaches.

Task	TCA-DNN	CORAL-DNN	LSTM-DANN	CADA	BDnet
FD001–FD002	55,763	44,963	2798	2122	3563.29
FD001–FD003	16,991	14,165	99,646	8415	2189.71
FD001–FD004	52,053	38,697	25,812	11,577	4003.32
FD002–FD001	3590	3393	638	351	474.32
FD002–FD003	23,071	16,511	8976	5213	2525.53
FD002–FD004	62,852	29,029	16,248	15,106	8131.71
FD003–FD001	4581	1341	3780	1451	2725.45
FD003–FD002	73,026	68,326	11,472	5257	5923.09
FD003–FD004	11,407	8583	9345	3219	4102.11
FD004–FD001	154,842	170,037	43,264	1840	3535.12
FD004–FD002	38,095	14,427	7723	4460	4070.49
FD004–FD003	6919	4613	1167	682	549.18

## Data Availability

The data presented in this study are available on request from the corresponding author.
